# New frontiers in atom probe tomography: a review of research enabled by cryo and/or vacuum transfer systems

**DOI:** 10.1016/j.mtadv.2020.100090

**Published:** 2020-07-10

**Authors:** I.E. McCarroll, P.A.J. Bagot, A. Devaraj, D.E. Perea, J.M. Cairney

**Affiliations:** a Australian Centre for Microscopy and Microanalysis, University of Sydney, Madsen Building F09, NSW 2006, Australia; b Department of Materials, University of Oxford, Parks Road, Oxford, OX1 3PH, United Kingdom; c Physical and Computational Sciences Directorate, Pacific Northwest National Laboratory, P.O. Box 999, Richland, WA 99352, USA; d Environmental Molecular Science Laboratory, Pacific Northwest National Laboratory, P.O. Box 999 Richland, WA 99352, USA; e School of Aerospace, Mechanical and Mechatronic Engineering, University of Sydney, NSW 2006, Australia

**Keywords:** Focused ion beam, Catalysis, Hydrogen, Deuterium, Organic, Cryogenic, Ultrahigh vacuum, Soft matter

## Abstract

There has been a recent surge in the use of cryo and/or vacuum specimen preparation and transfer systems to broaden the scope of research enabled by the microscopy technique of atom probe tomography. This is driven by the fact that, as for many microscopes, the application of atom probes to air- and temperature-sensitive materials or wet biological specimens has previously been limited by transfer through air at room temperature. Here we provide an overview of areas of research that benefit from these new transfer and analysis protocols, as well as a review of current advances in transfer devices, environmental cells, and glove boxes for controlled specimen manipulation. This includes the study of catalysis and corrosion, biological samples, liquid-solid interfaces, natural aging, and the distribution of hydrogen in materials.

## 1. Introduction

The continued advancement of many disciplines of science relies heavily on advanced microscopy, especially medical, soft matter, plant, materials, and geological science. However, many of the highest resolution microscopes require high-vacuum environments, limiting their application to materials that remain stable in a vacuum and ruling out hydrated samples such as soft biological matter unless it is fixed or cryogenically prepared. Moreover, modern microscopy workflows typically involve the examination of a specimen in several different microscopes, or the preparation of a specimen in one type of instrument and analysis in another. Transfer through air thus creates further challenges for the study of air-sensitive or cryo-frozen materials.

Atom probe tomography (APT) is a powerful microscopy technique that provides detailed three-dimensional (3D) maps showing the arrangement of atoms within nanoscale volumes of matter. APT has contributed to major advances in materials science, from alloy design [1] to the development of semiconductors [2],the dating of geological materials [3] and has even provided new information about the structure of hard biological samples such as teeth and bone [4]. As for many microscopes, the application of the atom probe to air- and temperature-sensitive materials or wet biological specimens has been limited by through-air, room-temperature specimen transfer.

Recently, there has been major development in cryogenic preparation processes to enable electron microscopy of biological samples [5]. The use of cryogenic stages enables focused ion beam (FIB) preparation of cryogenically frozen specimens for analysis in scanning electron microscopes (SEM) and transmission electron microscopes (TEM). In 2006, Marko et al. [6] provided direct evidence that vitrified specimens prepared by FIB could be transferred, without contamination, to a TEM, where it was confirmed by analysis of the crystal State that no significant warming had occurred during transfer. In a follow-up study, the same group was able to show that this technique could be applied to whole frozen-hydrated cells such as Escherichia coli (*E. coli*) [7]. Over the last decade, advanced cryogenic-based fabrication methods have evolved, becoming more standardized, and many options are now commercially available for the biological electron microscopy community [8].

Owing to the unique shape of an atom probe sample, the specific sample holder required, and the time-consuming insertion process through three vacuum chambers (required to ensure that the analysis chamber remains at ultrahigh vacuum (UHV), the developments within the biological sciences community are not directly transferrable to APT. Researchers hoping to use APT for air-and temperature-sensitive samples require transfer systems capable of transferring atom probe samples between the atom probe analysis chamber and another experimental platform, either under vacuum or under both vacuum and cryogenic conditions. Here we provide some background on recent atom probe work that has been conducted in the research areas that are benefiting from the development of vacuum and cryo transfer systems. This is followed by a review of the new technologies and the impact/potential impact they are having in these fields.

## 2. Key research drivers

There are a number of research areas that have been driving the development of environmentally controlled transfer between the atom probe and sample preparation and analysis platforms. Most prominent are catalysis, hydrogen embrittlement, and the study of biological matter. Substantial research has already been conducted in each of these areas using traditional non-cryo and non-vacuum transfer. We start by reviewing this background work before introducing the new technologies and the new projects that they have enabled.

### 2.1. Catalysis

Catalysis is an essential part of the world's manufacturing processes. Understanding the composition of surfaces and the reactions that take place at these surfaces in different chemical environments is essential for research into catalytic processes. The geometry of field ion microscopy (FIM) and APT samples is particularly advantageous for catalytic studies, exposing multiple crystallographic faces on a curved surface, providing a convenient close approximation for the multifaceted surfaces of catalyst nano-particles. This is in contrast to the more traditional surface-sensitive science studies that use single crystals, which do not permit examination of interplane diffusion or the role of highly stepped surfaces. This benefit has been exploited by FIM studies designed to investigate fundamental surface diffusion behavior, as well as how different gaseous/temperature exposures can cause reconstruction and/or segregation at the surface. The extensive literature on thermally activated diffusion kinetics of various individual adatoms within single crystallographic regions was reviewed by Kellogg in 1993 [9]. In related studies, researchers have also used APT to examine the mechanisms and kinetics of oxidation within the bulk of catalytically important alloys [10–14]. More recently, a study related to the stability of catalysts during the oxygen evolution reaction was undertaken using APT as the working electrode in an electrolytic cell [15].

In the late 1970s, Tsong et al. [16, 17] produced seminal works on catalytic materials by demonstrating the suitability of one-dimensional (1D) atom probe to carefully examine the surface and near-surface layers of individual crystallographic planes in various Pt-group alloys. These studies were greatly enhanced by the added capability to anneal specimens in vacuo or in the presence of trace gases such as oxygen and sulfur dioxide. They demonstrated very clearly that pronounced surface segregation can occur in binary and higher order alloys, with oscillations or monotonic fluctuations in composition visible within discrete atomic layers, highly dependent on environmental exposure conditions such as time, temperature, and the presence of even trace (parts per million) levels of chemisorbing species such as sulfur [18]. These works still stand as striking proof as to the need for nanoscale characterization of catalyst surfaces, where often the active surface layers have compositions that greatly deviate from bulk measurements or theorized values. The sensitivity of the surface chemistry to the environment underlines the need for the use of specialized reaction cell systems for controlled exposures.

### 2.2. Hydrogen

Understanding hydrogen in materials is extremely important to a number of industries. For example, the development of hydrogen as a clean energy source requires the development of hydrogen storage materials and fuel cells. Hydrogen plays an important role in catalysis and corrosion. Hydrogen also wreaks havoc in many alloy systems, leading to embrittlement that can cause catastrophic failure. For a comprehensive review of microstructural hydrogen mapping (with a focus on steels), the reader is referred to a 2017 review article by Koyama et al. [19].

However, understanding the distribution of hydrogen within material structures is an enormous challenge for materials scientists. Because the hydrogen atom is so light and mobile, it is almost impossible to observe directly with conventional microscopy techniques. Hydrogen is readily detected by APT, however, it is challenging to determine whether its origin arises from the specimen itself or from background hydrogen that is known to be present in the UHV chamber. To provide certainty about the origin of the hydrogen, samples can be charged with deuterium, the less common stable isotope of hydrogen (0.015% natural abundance). This approach allows the deuterium to serve as a marker for hydrogen, so that the location of the hydrogen atoms relating to the experiment can be determined unambiguously, noting that the chemical properties of hydrogen and deuterium are nearly the same, with a difference in the diffusion coefficient of deuterium in most materials being slightly lower due to its larger mass. This approach was first demonstrated in 2002 by Kesten et al. [20], by using APT to identify the location of deuterium in paladium/niobium and iron/vanadium multilayers. In this work, a depletion of deuterium in the vanadium and niobium layers was identified at the multilayer interface resulting from an intermixing of adjacent metal atoms, suggesting that the depletion of deuterium was a result of chemical intermixing. More recent research may suggest that this observation was a result of diffusion and/or ion trajectory aberrations induced during field evaporation [21–23]. Again in 2009, Gemma et al. [24] used APT to observe a high concentration of deuterium atoms in the vanadium layers of a deuterium-loaded iron/vanadium multilayer specimen. Haley et al. [25] were also able to show that a deuterium signal could be identified in two hydride-forming systems, palladium/rhodium and vanadium, by ex situ deuterium gas charging. However, the amount of deuterium detected differed significantly from theoretically predicted values and the specimens oxidized during transfer to the atom probe. They concluded that an *in situ* approach could reduce transfer times and would limit, if not eliminate, the possibility of oxidation.

### 2.3. Bioorganic and organic materials

#### 2.3.1. Hard biological matter

Although solid/hard biological materials, such as teeth, bone, and even certain proteins, do not commonly require special environmental or cryogenic transfer tools for atom probe analysis, two major developments were required to enable APT of these types of materials. First, the use of laser-assisted field evaporation, which allows for the study of non-conductive specimen [26], and second, the application of FIB methods for specimen preparation [27, 28], which make it possible to prepare needle-shaped specimen from solid biological samples with minimal damage.

**Fig. 1.**
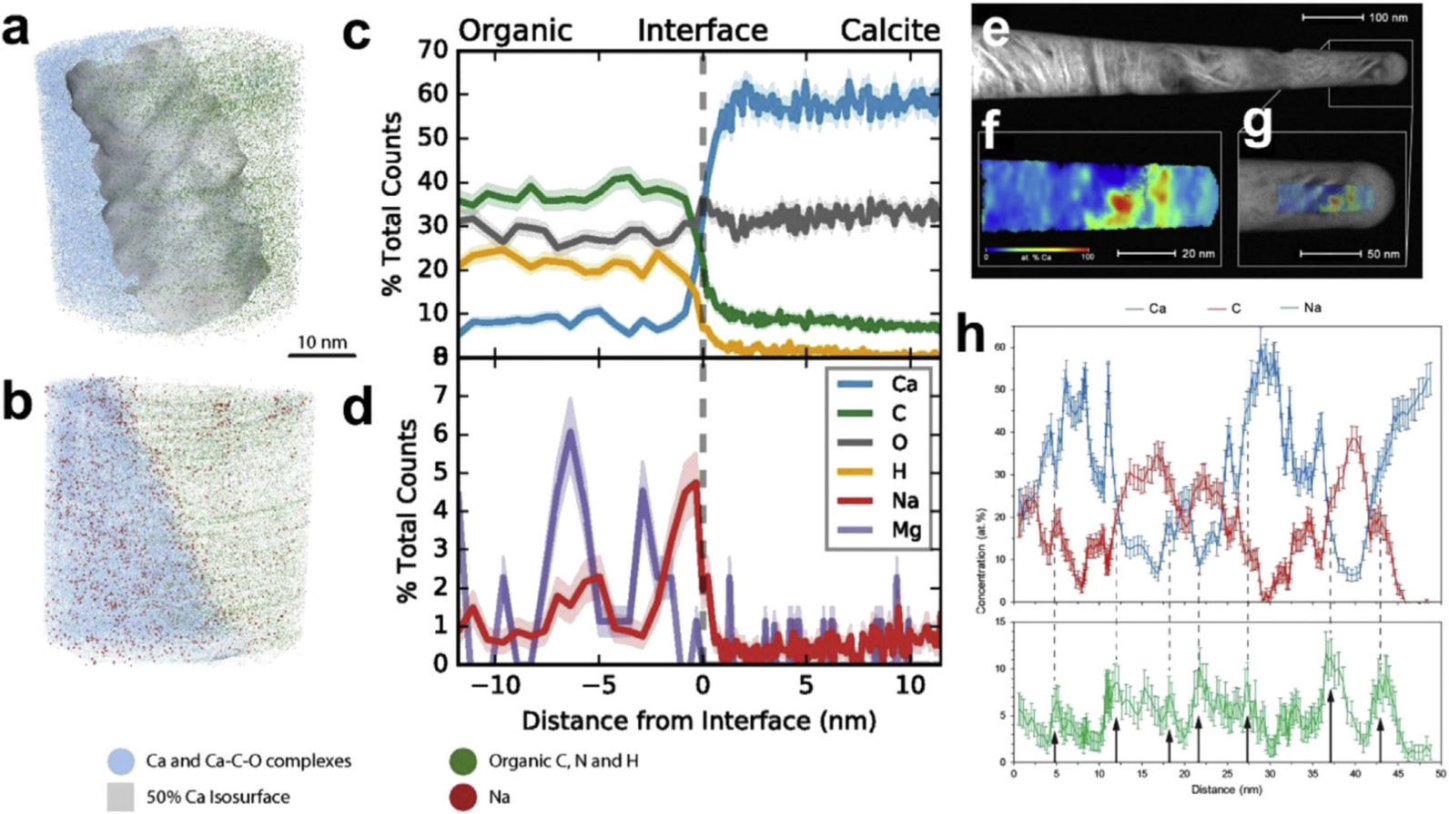
Atomic map of mineral-organic interfaces in (a—d) marine foraminiferal calcite showing the compositional portioning in segregation of Na to the interface [36] and (e—h) correlative microscopy of human bone [34]. (e) STEM image of the APT needle, (f) 2D map of the distribution of Ca in the specimen, (g) the overlay of STEM image and APT reconstruction, and (h) compositional profile showing the heterogenous distribution of minerals, organic components, and associated Na segregation. STEM, Scanning transmission electron microscopy.

A number of biomineralized materials, such as human and animal tooth enamel [29–32], chiton tooth [4], elephant tusk dentin [33], bone [34], fish scales [35], and marine foraminiferal calcite [36], have been successfully examined by APT. It has been possible to provide valuable insights into compositional heterogeneities at a nanoscale spatial resolution. For example, APT has revealed that dental enamel (rodent and human) contains a magnesium-rich amorphous calcium phosphate phase between the nanowires that make up the enamel structure [31]. Dental decay/caries has been shown to progress along magnesium-rich regions, mainly in the center of enamel nanocrystals [32]. APT has also helped decipher the microstructure-mechanical property relationships and proposed growth mechanisms within mouse incisor enamel, where the mechanical behavior was attributed to the presence of the magnesium-rich phase at grain boundaries, rather than organic phases [30]. Analysis of biological samples has provided new information about the arrangement of collagen fibrils as well as segregation of key elements such as sodium-to-organic calcite interfaces in marine foraminiferal calcite (i.e., the shells of marine organisms), as highlighted in Fig. 1 [34, 36]. More recently, APT was shown to be a promising method to examine the distribution of atoms in the hard biomaterials that are found in certain parts of insects such as the legs, jaw, teeth, claws, and stings of arthropods [37]. These structures are not biomineralized; instead, they are predominantly organic but enriched in heavy elements (e.g. zinc, manganese, and copper). It was shown that the zinc in ant teeth is evenly distributed and that the Zn is incorporated gradually over the life of the species, with more zinc being observed in adults than in juveniles.

#### 2.3.2. Polymers

Prosa et al. [38] studied conducting poly(3-alkylthiophene) polymers deposited on presharpened Al tips. The purpose of this study was to better understand the applicability of APT to organic materials, and it was suggested that these polymers might be able to act as host materials for embedding other materials (organic/ biological materials or nanoparticles). Two methods were developed for application of the polymer film on the tip. In the first, a tip was dipped in a polymer solution and the solution was allowed to evaporate leaving behind a coating of polymer. The second utilized electrospray ionization deposition. In both cases, molecular fragments were detected, rather than single atoms. In order of typical relative abundance, these fragments were identified as $C_{2}H_{5}^{+}$, ${CH}_{3}^{+}$, $C_{2}H_{4}^{+}$, followed by $C_{3}H_{7,8}^{+}/SC^{+}$ and SCH^+^.

#### 2.3.3. Cellular structures

Most soft biological materials (e.g., cellular organelles, proteins, etc) exist within a hydrated environment, making their preparation and analysis in the high to ultrahigh vacuum environment of the FIB/SEM and the atom probe challenging. Literature relating to the examination of soft, organic matter is thus currently limited. In 2012, researchers explored the potential of APT to map the structure of unstained, freeze-dried mammalian cells [39]. Peaks corresponding to C, Na, and K ions showed distinct patterns of spatial distribution within the cells, indicating the potential of APT for mapping the subcellular distribution of atomic species, such as labeled metabolites. In 2016, Adineh et al. [40] examined a polymyxin-susceptible strain of *Acinetobacter baumannii* gram-negative bacteria, a so-called ‘superbug’. After culturing, the bacterial cells were fixed in 4 % paraformaldehyde in phosphate-buffered saline (PBS) and air-dried on silicon nitride membranes. Needle-shaped specimens were prepared by FIB at room temperature and a silver coating was applied. Data presented was identified as being from the intracellular domain and cell envelope regions of the bacterial cell. Although the emphasis of this work was on the demonstration of the methods for atom probe examination, the distinct mass spectra from drug-susceptible and drug-resistant strains, primarily at the cell envelope, were able to shed light on the compositional changes involved in the development of drug-resistant mechanisms. The work by Adineh et al. relied on fixed biological samples to create a bulk solid specimen using conventional FIB. Chemical fixation is a common process to preserve the structure of biological samples in resin for imaging slices (histology).

The chemical fixation of biological samples in organic resins is not ideal [41]. Firstly, chemical fixation can alter the sample on a molecular level and can create artifacts that interfere with interpretation of the cellular ultrastructure and destroy the distribution of ionic species. Secondly, the organic resin matrix of the chemical fixative is difficult to distinguish from the bioorganic specimen because the resin and biomaterial are made of the same elements. These issues have been a driving factor in the development of cryo-based specimen preparation methods for electron microscopy analysis of bioorganic structures ‘frozen’ in their native state. By retaining water ice, instead of replacing it with the chemical fixative organic resin, it may be possible to distinguish the bioorganic materials, potentially opening up the technique of APT to direct mapping of the macromolecular structure and ionic gradients in native biological systems.

To minimize or eliminate structural artifacts in cryo-prepared specimens in water ice, great care must be taken to properly freeze specimens. In specimens of sufficiently small volume, rapid plunge freezing of aqueous solutions causes them to transform directly to a vitreous ice phase that preserves the original structure, avoiding the damage that normally occurs during volume expansion when crystalline ice forms during conventional freezing. The first thermally assisted field ionization and time-of-flight studies on aqueous KCl in a vitreous state on tungsten and gold surfaces were carried out by Stintz and Panitz in 1991 [42]. Images of the desorbing ions showed no order in the ice layer on a nanometer length scale, and it was concluded that the ice was vitreous. They achieved the rapid cooling of the fluid to cryogenic temperatures by plunging the specimen tip into a container of liquid propane. Once cooled, the tip was transferred to the atom probe using a cooled anode assembly that completely encapsulated the tip[43, 44].

More recently, Adineh et al. developed a unique approach to sandwich biological materials (e.g., metal nanoparticles and bacterial cell culture) between a tungsten needle tip and a graphene coating [45]. This was accomplished by lowering a small-diameter metal ring with a suspended droplet of biomaterial sample with a graphene film floating on top, over a tungsten needle tip. Importantly, they report that the mass resolving power is significantly enhanced by the graphene coating, due to the improvement in the tip conductivity, which led to improved compositional accuracy upon analysis of the 3D data. This method was used to sandwich a water nanomembrane (WNM) onto the apex of a tip. The presence of such a layer was confirmed by transmission electron microscopy. The impermeability of the graphene prevents the water from evaporating or subliming in the vacuum. It is possible that such a sample could be host to biological (or other) specimens for analysis. Despite the graphene preventing the desiccation of the specimen under the high to ultrahigh vacuum conditions of the SEM, TEM, and APT tools, it is unknown how the slow freezing of the specimen in the APT analysis chamber would affect the specimen (i.e. vitreous or crystalline ice formation). We envision that a possible addition to the workflow would be to rapidly cryogenically freeze the graphene-encased sample ex situ followed by environmentally protected transfer to the atom probe analysis chamber.

#### 2.3.4. Biomolecules

The field of structural biology is dedicated to revealing the 3D structure of proteins and how they relate to their function in cells. This information helps to understand how certain proteins interact with each other, with the ultimate goal of using the knowledge of the 3D structure of critical proteins to develop new treatments for disease. Until recently, x-ray crystallography was the primary method for determining 3D structures, however, cryo electron microscopy is rapidly becoming the tool of choice for these studies [46]. Single-particle cryo TEM can be used to determine the 3D structure of individual molecules, such as proteins in solution, but these structures can vary when the proteins are in their native state within tissue. Cryo tomography techniques such as TEM tomography and sequential cross-sectioning methods that use a FIB or a microtome, provide 3D structural information and can even be correlated with data from light and optical microscopy techniques to identify the location of specific biomolecules [47]. However, these techniques do not provide the resolution of the single-particle methods. To be able to identify the structure of proteins (or other molecules) with APT would be revolutionary, but the mass spectral resolution of APT coupled with a relatively low analysis yield are issues that pose major challenges, even if cryo samples were successfully prepared, transferred, and analyzed.

Early attempts to image individual biological molecules by field emission technologies [48–50], motivated by the high image contrast, magnification, and resolution of field emission techniques, have been reviewed by Kelly et al. [51] and were largely unsuccessful. The first correlated, and thus verifiable, images of biomolecules were made in the early 1980s [52–55] in afield desorption point projection microscope. Ferritin molecules on a tungsten tip, identified in a TEM by their iron-rich center, were correlated to the point projection images [52]. Following on from the initial success of the ferritin studies, this research was expanded to include the successful study of unstained nucleic acids [56]. Although this visualization method was able to provide the expected high contrast, magnification, and resolution, it was not able to provide any new information relating to the composition of the molecules.

**Fig. 2.**
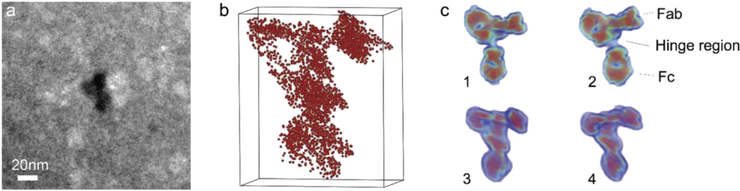
a) TEM micrograph showing the IgG within the solid silica matrix and b) APT reconstruction of a single IgG molecule showing the spatial distribution of ${CNH}_{2}^{+}$ and $CO_{2}^{+}$ c) carbon isodensity heat maps derived from two different techniques 1 and 2: human IgG derived from X-ray diffraction [59] and 3 and 4 rabbit IgG derived from APT [60].

More recently, Perea et al. and Sundell et al. have separately reported on atom probe analysis of individual protein molecules embedded in a solid organic and inorganic matrix, respectively. Perea et al. reported on the composition of individual ferritin protein molecules providing an average radial composition to reveal an Fe-rich mineral core, surrounded by a bilayer rich in P followed by a layer rich in Na [57]. The results are consistent with previous indirect determinations of P-enriched surfaces of mammalian ferritins. The results demonstrated a viable application of APT to study complex biological interfaces. Although this was the first report of APT being used to directly measure the 3D composition of an ensemble of individual proteins, the composition of the organic resin matrix prevented the unambiguous distinction from the proteins. Similarly, Sundell et al. [58] examined a well-characterized antibody, rabbit immunoglobulin G (IgG), by using a sol-gel method to embed individual proteins in an amorphous solid silica matrix, followed by a standard FIB liftout to prepare the required needle-shaped specimen. The resulting data did not contain the characteristic peaks of water at 17, 18, and 19 Da, suggesting that the hydration shell around the molecule was completely replaced with silica during the condensation process (H and O were present throughout the analysis, attributed to contamination, meaning that the presence of water could not be ruled out). By importing the reported structural data for IgG into the atom probe data analysis software, it was possible to prepare comparison heat map images of the atomic density of carbon between the reported structure and the atom probe data set (Fig. 2). Even with the use of an atom probe limited to a detection efficiency limit of 37 %, the 3D reconstructions showed good agreement with the protein databank IgG crystal structure, with key structural details of the protein visible.

## 3. Technology development

It is clear that APT is useful for research related to catalysis, hydrogen embrittlement and organic materials. However, it has also been established that to harness its full potential within these research fields, advances to sample preparation and sample transfer are required. Over the years, significant technological advances have been made in advanced sample preparation and transfer, and new applications of APT are now being explored [61]. Two classes of transfer devices have emerged: 1) coupled devices and 2) shuttle transfer systems. Coupled devices refer to direct adaptations affixed to the atom probe, i.e. where specimen are directly transferred from an experimental device, such as a gas-phase reaction cell, to the atom probe. Such coupled/in situ devices provide a conceptually simple and rapid transfer solution, providing ease of use and minimization of surface contamination during transfer. However, they limit the analysis to within the confines of the system (i.e., the experimental device and the atom probe itself). Shuttle systems refer to the integration of various microscopy techniques and experimental platforms through the use of a specimen shuttle/suitcase-style transfer device, which can be held under vacuum and/or cryogenic conditions. Recent technological developments and the studies enabled at each stage of development will be reviewed in this section.

### 3.1. Developments in coupled transfer systems

Following on from the early catalytic studies, Kruse et al. led an ongoing effort in the application of APT to catalysis. Over a number of years, Kruse group et al carried out unique studies that involved imaging Pt-group metal specimens by FIM, while simultaneously exposing them to low pressures (~10^−3^ Pa) of reactive gases, such as nitric oxide, at elevated temperatures (~500 K) [62–65]. FIM studies, exploring the hydrogenation of NO and NO2 over Pt and Pd surfaces using field emission techniques, continue to be carried out by the Brussels-based group [66]. Direct exposure of the analysis chamber to high temperature/pressure is not suited to modern instruments, which require UHV environments to collect data. Research in the field of catalysis has therefore shifted to ‘post-mortem’ studies, where specimens are exposed to specific environments in a separate reaction cell system, physically connected to the atom probe (i.e. a coupled device). After specimens are exposed to reactant gases under controlled pressure and temperature, the reactor is evacuated to high vacuum (HV) or UHV and the specimens are transferred to the analysis chamber for analysis. The catalytic atom probe (CAP), developed at Oxford University by Bagot et al. [67] in 2006, was the first example of a postmortem-style system, consisting of a reaction cell attached to a 3D atom probe (3DAP). The CAP better approximated real-world exposures of heterogeneous catalytic materials by allowing exposure to gaseous atmospheres at greater pressures (≤1 bar) and at higher temperatures (≤873 K). The CAP enabled a number of studies on surface segregation trends in binary [68] and ternary [69] Pt-group alloys, as well as Au and Ag alloys [70, 71]. Through studying the Pt-group alloys by using the CAP, it was determined that the influence of Ru or Ir additions on the surface behavior was dependent on which crystal faces were exposed to the NO gas. This indicated that controlling which crystal faces are exposed to the reaction gases could improve the efficiency of the catalyst particles. The study of Au-Pd alloys revealed that exposure of these systems to NO gases at temperatures >300 K induced segregation of Pd towards the surface of the alloy. No segregation was observed on alloys prior to gas and temperature exposure, contrary to predictions made by Metropolis Monte Carlo simulations, which predicted Au segregation at thermal equilibrium. This study revealed that the surface concentration of Pd could be controlled through exposure of Au-Pd alloys to NO gases under appropriate thermal conditions.

The application of CAP was not limited to catalysis studies. In 2010, Takahashi et al. [72] at Nippon Steel in Japan were the first to use this same approach to provide direct observation of deuterium, as an indicator for hydrogen, at trapping sites in alloys (i.e. precipitates in steels). They achieved this by attaching a ‘deuterium charge cell’ to the storage chamber of a 3DAP. Incorporated into this particular transfer system was a liquid nitrogen cooled cold finger, capable of cooling specimens to <173 K within seconds. Direct charging of electropolished needles was performed in a deuterium gas atmosphere of 0.8 atm. Because the solubility of hydrogen at 1 atm is very low at room temperature (<0.1 wt%) and there are high-potential barriers (~100 kJ/mol) at the steel surface, the tip of the specimen was locally heated to 523–573 K to increase the hydrogen solubility and overcome surface barriers. Specimens were charged for 5–10 min, and then the heater was removed from the tip position and the gas pumped out of the cell. The specimen was rapidly cooled (within a few seconds) and kept below 173 K while it was transferred to the analysis chamber (below 70 K). It was less than 3 min between the heater removal and the insertion to the analysis chamber. Using this approach, Takahashi et al. [72] were able to show that hydrogen becomes trapped at titanium carbide precipitates in steel. This same approach was later used to demonstrate that vanadium carbides also act as hydrogen-trapping sites [73, 74].

To build on the capabilities of the early CAPs, researchers at Iowa State University, in collaboration with researchers from Oxford University, developed the first reaction cell to be attached to a local electrode atom probe (LEAP) instrument [75]. Initial experiments using the 3DAP were limited to voltage-pulsed acquisition and provided only a small field of view. By attaching the new reaction cell to a LEAP instrument, experiments could utilise advances in APT including laser pulsing mode and a wider field of view [76, 77]. The ability to apply a laser pulse to reaction experiments in the new instrumentation expanded the field of research from catalytic surface reactions to include the study of non-conductive materials, such as materials that had developed large oxidation layers during more aggressive gas exposures. Initial results generated from the reaction cell included studies of the early stages of oxidation of an Al alloy and surface reactions on Pt alloys, demonstrating the capacity for this technique to capture the onset of oxidation on multiple materials [75].

**Fig. 3.**
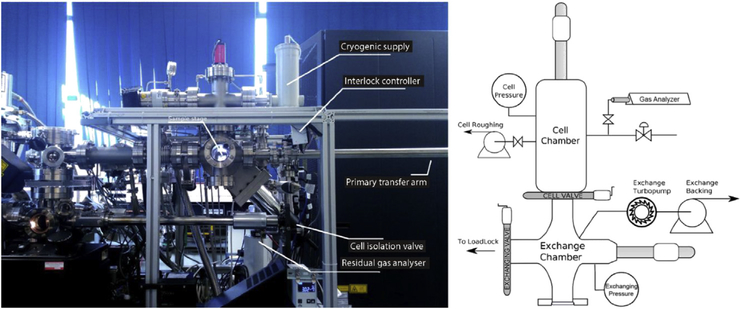
Modern reaction cell integrated onto a LEAP 3000X-HR, adapted from the study by Haley et al. [78].

Recently, a gas-phase reaction cell was designed at Oxford University to interface with a LEAP 3000X-HR [78] (Fig. 3). This system combined the capabilities of the CAP and the ‘deuterium charge cell,’ including the capacity to expose a tip to a variety of gases at or below atmospheric pressures and the capacity for gaseous deuterium charging followed immediately by cryogenic quenching. The stage assembly is equipped with an electrically resistant cartridge heater capable of heating a specimen up to a temperature of 723 K. The main chamber used for gas exposure is connected via an isolation valve to a residual gas analyzer, providing confirmation as to the quality of the vacuum before gas exposure. This system was designed primarily for the analysis of H uptake in various metal systems, and its effectiveness for the analysis of H uptake was validated using near-pure Pd (99.95%) under deuterium gas exposure. This system was also used to study the onset of oxidation of an Mg alloy [79]. Mg alloys were electropolished into needle-shaped specimens, they were then directly transferred to the atom probe vacuum system. All specimens were initially analyzed by APT to remove any oxide that may have developed during sample preparation. After initial removal of the oxide, specimens were transferred to the reaction cell where they were exposed to O_2_. The results indicated that the onset of oxidation of the Mg alloy was affected by the presence of H within the sample prior to gas exposure, validating a theory related to the mechanisms of Mg oxidation that had been almost disregarded owing to the lack of supporting evidence [79].

### 3.2. Developments in shuttle transfer systems

Coupled/in situ devices provide a simple and rapid transfer solution, providing ease of use and minimization of surface contamination during transfer. However, these systems are limited in their application as they cannot be used in correlative microscopy studies, and no systems of this kind were designed that were able to be applied to the preparation of tips from soft matter. These studies require new hardware technology and protocol workflows that enable the transfer of specimen between different devices, e.g. between a glove box and an atom probe, or a FIB and an atom probe, under controlled conditions. In 2015, Gerstl and Wepf [80] developed the first APT-specific cryogenic shuttle transfer system at ETH-Zurich. Their transfer system enabled vacuum-cryo-transfer (VCT) between a cryo-modified FIB/SEM and a LEAP.

#### 3.2.1. Materials science applications

An early application of the ETH Zürich shuttle system was to investigate precipitate formation during natural aging of Al-Mg-Si alloys [81]. Natural aging processes that occur in Al alloys before artificial aging are thought to negatively impact the mechanical properties of the final alloy. Unfortunately, these processes occur rapidly, sometimes in a matter of minutes, and require cryogenic cooling to literally freeze the natural aging processes at a given time to explore the evolution of the microstructure as the natural aging cycle progresses. In a conventional experiment, the time required for the specimen to be transferred from the sample preparation environment to the UHV analysis chamber (which is cryogenically cooled) is on the order of an hour or more, well beyond the time required for natural aging to occur. Through use of the VCT, Dumitraschkewitz et al. were able to reduce the time spent at room temperature to approximately one minute [81]. As a result, they were able to experimentally verify that clusters of Si are the first to form during the natural aging process. In a later study, the same team also used the same approach to demonstrate that specimen size–dependent diffusion in atom probe tips affect the aging and clustering processes, an important consideration for the study of microstructural changes via microscopy techniques [82].

The VCT at ETH has also been utilised in studying the role of precipitates in resisting the effects of hydrogen embrittlement in steels. In a study by Chen et al. [83], a ferritic steel with dispersed V-Mo-Nb carbides was electrochemically charged with deuterium using a solution of 0.1 M NaOD in heavy water (D_2_O). After charging, the specimen was rapidly cooled to cryogenic temperatures and transferred via the VCT to the atom probe for analysis. The results of this study clearly showed a concentration of deuterium at the V-Mo-Nb precipitates, providing direct evidence that hydrogen is trapped within these carbides.

In an initial attempt to develop controlled transfer of atom probe samples at the University of Sydney, Eder et al. developed a glove bag transfer system for application to the study of self-assembled monolayers (SAMs) [84]. SAMs are attractive materials in the field of nanofabrication and nanotechnology owing to their surface modification capacity [85]. Gault et al. [86] had previously used atom probes to study whether adsorbed species were randomly adsorbed molecules or whether these molecules represented an organized SAM. This initial research also provided evidence as to the state of disassociated S-H bonds during formation of the SAM. Earlier, similar research had been undertaken by Zhang and Hillier [87] to show the applicability of APT to the study of adsorbed monolayers. Zhang and Hillier used a gold substrate due to its naturally inert nature, enabling the adsorption to occur without concern for surface changes induced by oxidation during specimen transfer. However, gold does not run well in the atom probe, creating uncertainties in the reconstructed data. Similar issues with gold substrates were observed by Stoffers et al. [88]. The study by Eder et al. [84] explored the adsorption of thiophene on various substrates, including aluminum, platinum and tungsten. The results indicated a substantial variation in the way thiophene adsorbs to the substrate surface. It was also noted that an oxide which formed on the surface of the tungsten had a strong effect on the adsorption behavior. To make a comparison between the three non-oxidized surfaces, it was necessary to use the glove bag transfer system to enable dipping and transfer of the tip in an inert environment, preventing oxidation. A comparison between ambient air transfer and glove bag transfer for a tungsten specimen proved the efficacy of the glove bag system by demonstrating the development of a layer of tungsten oxide on the surface during the ambient air transfer and an oxide-free surface for the specimen transferred via the glove bag. Results from a variety of substrates, Al, Pt, W, and WO, indicated a substantial variation in the way the thiophene was adsorbed, providing valuable information about the distribution, density, and even desorption energy of thiophene molecules on these different surfaces.

Motivated by the need for inert transfer of specimens to study SAMs, a purpose-built glove box for environmentally controlled exposures was installed at the University of Sydney. The glove box operates under a dry nitrogen environment with relative humidity levels approaching zero. It is designed to be compatible with the Ferrovac VCT system [89] and has the capacity to facilitate specimen exchange between the VCT and a TEM holder without breaking the cryo chain. The VCT system currently links the glove box with a Ga-ion FIB/SEM and a LEAP 4000X Si under UHV and cryogenic conditions. The transfer system is actively pumped using an ion pump capable of reaching vacuum levels of 10^−10^ mbar and actively cooled by a dewar filled with liquid nitrogen, reaching a stage temperature of 185 °C. The FIB/SEM is equipped with a cryo-cooled stage, reaching stable temperatures of <145 °C, with full tilt capacity and ~180 ° of rotation.

The first application of this integrated cryo-suite was to investigate deuterated alloys to better understand hydrogen embrittlement [90]. Presharpened steel tips were electrochemically deuterated, cooled to cryogenic temperatures, and transferred to the atom probe for analysis of the deuterium distribution, without frost accumulating on the tip. Deuterium was observed at different microstructural features within the steel specimen. For the first time, hydrogen was observed at dislocations in a martensitic steel, supporting the theory of hydrogen-enhanced dislocation mobility as a mechanism of hydrogen embrittlement. Observations of hydrogen at an incoherent interface between niobium carbides and the surrounding steel provided the first direct evidence that these incoherent boundaries can act as trapping sites. Attempts at transferring deuterated martensitic steels via alternative methods, i.e. non-cryo-integrated, failed to maintain the deuterium at trapping sites, further validating the need for cryo-integrated systems in the study of hydrogen embrittlement.

Researchers at the Max-Planck-Institut für Eisenforschung in Dusseldorf also used a Ferrovac VCT system to transfer environmentally sensitive samples between an LEAP 5000 XS and XR, a Xeplasma FIB/SEM, and a Syletec glove box modified to enable specimen transfer to the VCT system [91]. To highlight the relevance of a cryo-integrated equipment suite, Stephenson et al. [91] compared the MgO, Mg_2_O, and O distribution on pure Mg with various transfer combinations: room temperature FIB and transfer, cryo FIB and room temperature transfer, cryo FIB and cryo transfer. They found that surface oxidation was suppressed by using an entirely cryogenic protocol, confirming the importance of using cryo transfer to eliminate oxidation of the tip during transfer from the FIB to the atom probe. Previously it has been shown that the use of FIB preparation methods can induce the formation of hydrides in Ti alloys [92]. Chang et al. [93] compared APT and TEM specimens prepared at room temperature and under cryogenic conditions. Both APT and TEM results confirmed that the formation of hydrides caused by FIB sample preparation could be eliminated by cryo preparation.

For energy storage materials, specifically Li-, Na-, or Mg-ion battery electrode materials, it is critical to be able to analyze the distribution of the light elements. Vacuum transfer capabilities at the Pacific Northwest National Laboratory (PNNL), USA, have been used to allow visualization of the Li distribution in battery electrode materials and provide information about the degradation pathways through examination at different extents of cycling [94, 95]. The cycled electrodes are highly reactive with atmosphere, and to ensure accurate analysis of such materials, cryogenic specimen preparation in FIB and transfer to APT using VCT will be required to avoid any unintentional environmentally induced modifications.

#### 3.2.2. Organic material applications

**Fig. 4.**
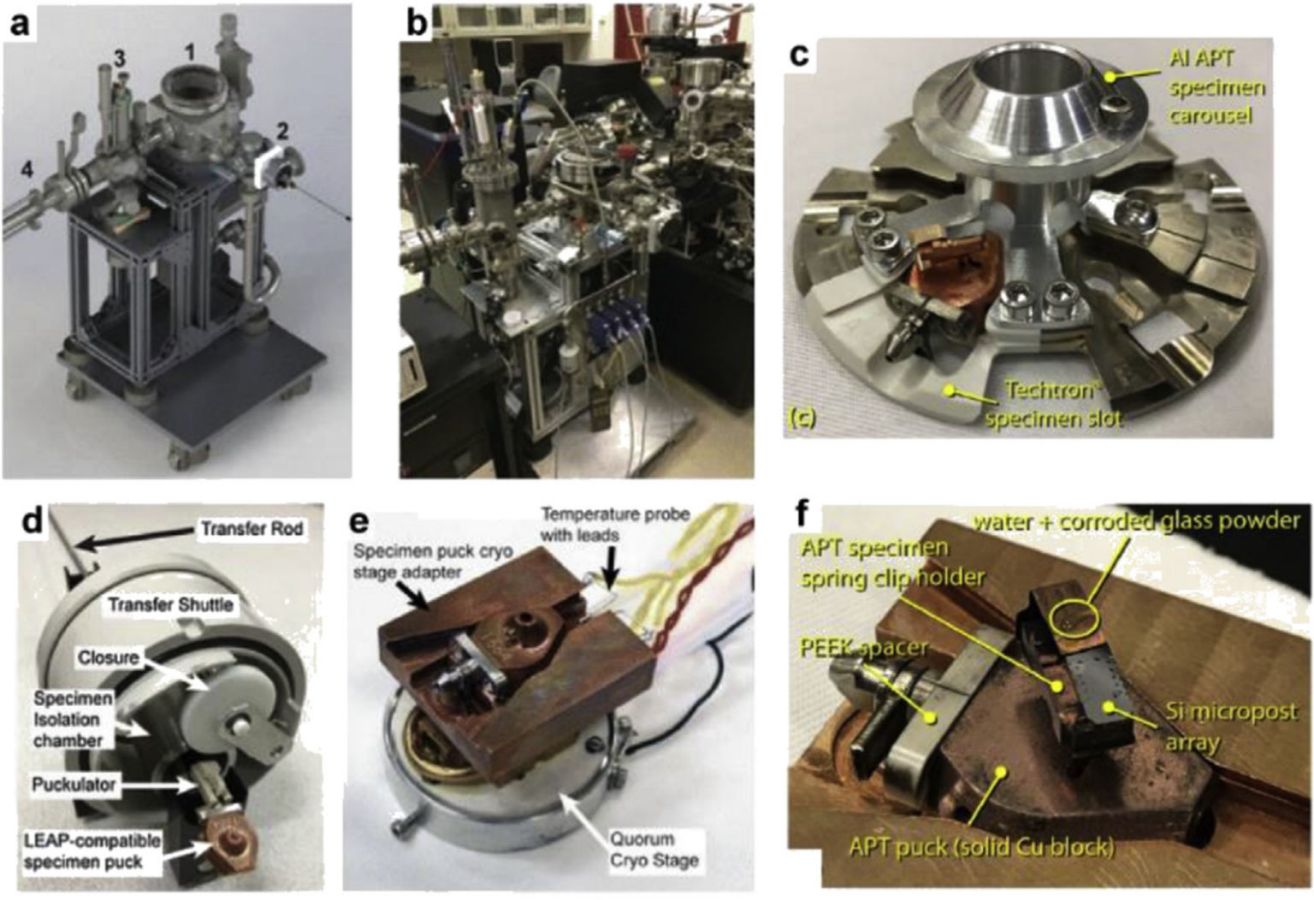
Custom hardware technology to enable FIB-based preparation and specimen-protected transfer of environmentally sensitive materials between a FIB/SEM and the LEAP at the Pacific Northwest National Laboratory (PNNL; WA, USA). (a) CAD-rendered image of the environmental transfer hub (ETH) main parts as (1) main vacuum chamber hub, (2), docking port for specimen transfer shuttle, (3) high-temperature ambient pressure reactor chamber, and (4) manipulator to transfer specimens between the ETH and the LEAP. (b) Photograph of the ETH system connected to the atom probe at PNNL. (c) Puck carousel with a modified APT puck slot made from thermally insulating material. (d—e) Modified environmental shuttle suitcase and FIB/SEM cold stage, respectively. Modifications were made to handle the transfer and manipulation of (f) the specific pucks used for APT analysis. Panels (a—b) and (d—e) are reproduced with permission from Perea et al. [96]. Panels (c) and (f) are reproduced with permission from Schreiber et al. [97]. PEEK, Poly-etheretherketone; CAD, computer aided design.

At the PNNL, Perea and Evans have developed a custom UHV chamber system, which they call the environmental transfer hub, or ETH (not to be confused with the ETH-Zurich institution in Switzerland). They have also modified the cryo-stage and specimen shuttle suitcase of a Quorum PT2010 cryo-FIB/SEM specimen preparation station to accept atom probe sample holders (pucks) (Fig. 4)[96, 97].

**Fig. 5.**
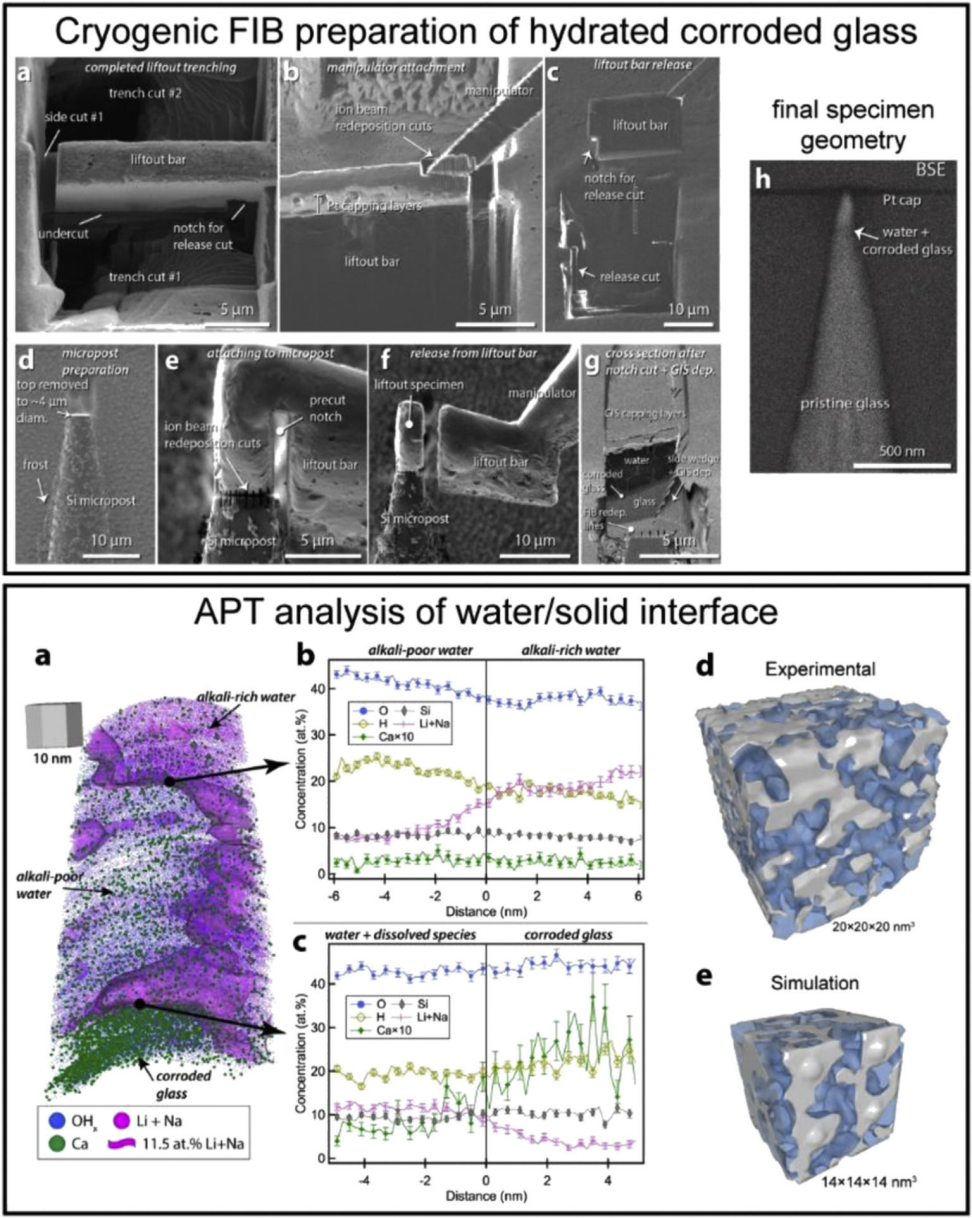
Top: FIB-based site-targeted preparation of a cryogenically frozen-hydrated corroded glass specimen. (a—b) SEM image of a trenched liftout bar and subsequent attachment of the micromanipulator. (c) Extracted bar after release cut. (d—f) Preparation, attachment, and release of a piece of the liftout bar onto Si micropost. (g) Cross section of a mounted specimen showing relevant layers. (h) Final specimen needle geometry with targeted water/solid interface highlighted. Bottom: APT analysis of water/solid interface of a corroded glass. (a) 3D APT atom map and composition profiles across (b) the alkali-rich/alkali-poor interface in water and (c) across the water/corroded glass interface. (d) Experimental and (e) simulated 3D network of water (blue) interpenetrating the silica nanoporous network (gray) in the corroded glass region. All figures reprinted with author permission: Top (a—h) and bottom from (a—c) the study by Schreiber et al. [97] and bottom (d—e) from the study by Perea et al. [98].

Using the ETH, Schreiber et al. [97] have developed a method to prepare APT needles from a cryogenically frozen, hydrated, corroded glass sample, with the innovation being the ability to target a buried water/solid interface, as described in Fig. 5-top. The subsequent analysis enabled by the environmentally protected transfer of the FIB-prepared specimens shown in Fig. 5 – bottom [98] reveals the tomographic compositional distribution of dissolved ions within the corroding water solution and the corroded glass network, as well as the 3D structure of the water-filled nanoporous corroded glass network. Although the results demonstrate a rational means to prepare, environmentally transfer, and analyze site-targeted cryogenically frozen specimens by APT, there are many challenges that inhibit the wider application of this approach. Some of these challenges are 1) preventing the sublimation of water ice when exposed to a high-energy Ga-ion beam and 2) lack of a reliable means to attach the FIB-prepared liftout to micro-posts under a cryogenic environment, where the currently available organic and metalorganic vapors fail to provide a means to controllably deposit thin films. Ultimately, continued advancements in the preparation and analysis of cryogenically prepared APT specimens is expected to lay the foundation for APT to impact our understanding of hydrated materials systems related to materials science, geochemistry, and biology.

Other environmentally protected specimen transfer systems exist at a number of different universities, including at Erlangen/ Nurnberg [99], University of Michigan, and the University of Oxford, and work from these institutions is forthcoming. All are configured for environmentally controlled atom probe experiments that require the manipulation, handling, and transfer of specimens using the puck carrier that is compatible with LEAP instruments. Notably, the Erlangen system is also compatible with a home-built atom probe system that is currently in development.

## 4. Outlook

Vacuum and cryogenic preparation and transfer tools for microscopy are evolving rapidly and it is now possible to apply these methods to the preparation of specimen for APT. This has the potential to allow a number of exciting new experiments that were not previously possible:

- The composition of surfaces during chemical interactions or reactions
- Measurement of the arrangement of molecules at hydrated surfaces (ligands)
- Compositional mapping across liquid-solid interfaces
- Observation of the position of hydrogen atoms by using deuterium as a tracer
- Atomic-scale mapping of ions or molecules in vitrified biological materials
- Direct observation of the arrangement of ions in vitrified liquids
- The analysis of highly reactive samples that are sensitive to air, such as battery or nuclear materials
- In some cases, successful FIB preparation of specimen that are affected by the ion beam at room temperature

For many of these examples, successful experiments will require substantial developments in both the experimental setup and the available tools for data treatment and analysis. The workflows required will involve the following:

- Effective protocols for cryogenic specimen preparation, gas exposure, deuteration, and/or vitrification and fabrication into the required needle-shaped samples
- Reliable transfer tools to ensure the pressure and temperature requirements are maintained throughout all steps from specimen preparation, transfer, and analysis
- Optimization of atom probe acquisition parameters and an understanding of how they affect the data, especially in systems that contain materials of vastly different evaporation fields
- Protocols for the interpretation of atom probe data from materials with little existing literature (e.g. organic materials)
- New reconstruction algorithms that can account for molecular ions (i.e. fragments)

In materials science, considering current interest in the field, it is reasonable to expect that deuterium labeling might become a standard method for measuring the distribution of hydrogen within materials. Hydrogen-free instruments, if successfully developed, may still require cryogenic transfer systems to ensure that hydrogen from charging remains within alloys. Cryo FIB instruments may prove useful to minimize FIB-induced damage and vacuum/cryo transfer to both atom probe and TEM is highly likely to become commonplace to avoid exposure of specimen to air.

We are likely to see the continued use of ancillary environmental chambers to study corrosion and catalysis. These are sociogenically important problems. Corrosion has an enormous economic impact. Evolving cryogenic specimen preparation and environmental transfer strategies will continue to allow APT analysis of corrosion product and across the liquid-solid interface [100], allowing for the design of corrosion mitigation strategies. Catalyst design for the conversion of biomass-based feedstocks to liquid fuels is one of the most promising alternatives to our dwindling fossil fuel reserves. Understanding the reactions at surfaces in different chemical environments is essential for this work.

There is scope for vacuum-based transfer methods to enable transformational research into battery materials, such as Li-based anode materials [101], and early results are indicating that cryo-stages will enable successful FIB preparation from materials that are sensitive to the ion beam, such as aluminum and unstable alloys systems.

It is difficult to predict the impact that atomic-scale imaging may have on life sciences. FIB-based site-specific specimen preparation and correlative light electron microscopy [102] might allow specific regions to be identified within biological structures, allowing the study of the distribution of biologically important elements such as Ca, Fe, N, Se, or foreign species such as heavy metals in relation to certain sub-cellular components. Staining or isotopic labeling might help to identify regions of interest. These approaches are well established for correlative light and electron microscopy. The wider impact of the technique will depend on whether biomolecules can be identified from the fragments that form during field ionization and/or the effectiveness of methods to label specific regions of interest. If it is possible to identify specific biomolecules, it would open up a whole new field of nanoscale analytical imaging in structural and molecular biology with applications ranging from plant science, to drug discovery, bioengineering, and medicine. Successfully applied, these new methods have the potential to lead to radical new discoveries in fields such as medicine, chemical processing, and agriculture, with flow-on benefits related to health, the economy, the environment, and our quality of life.
